# The use of genomic signature distance between bacteriophages and their hosts displays evolutionary relationships and phage growth cycle determination

**DOI:** 10.1186/1743-422X-7-163

**Published:** 2010-07-17

**Authors:** Patrick Deschavanne, Michael S DuBow, Christophe Regeard

**Affiliations:** 1 Molécules thérapeutiques in silico (MTI), INSERM UMR-M 973, Université Paris Diderot - Paris 7, Bât Lamarck, 35 rue Hélène Brion, 75205, Paris Cedex 13, France; 2 Université Paris-Sud XI, Institut de Génétique et Microbiologie, CNRS UMR 8621, Bâtiments 409, 91405 Orsay, France

## Abstract

**Background:**

Bacteriophage classification is mainly based on morphological traits and genome characteristics combined with host information and in some cases on phage growth lifestyle. A lack of molecular tools can impede more precise studies on phylogenetic relationships or even a taxonomic classification. The use of methods to analyze genome sequences without the requirement for homology has allowed advances in classification.

**Results:**

Here, we proposed to use genome sequence signature to characterize bacteriophages and to compare them to their host genome signature in order to obtain host-phage relationships and information on their lifestyle. We analyze the host-phage relationships in the four most representative groups of Caudoviridae, the dsDNA group of phages. We demonstrate that the use of phage genomic signature and its comparison with that of the host allows a grouping of phages and is also able to predict the host-phage relationships (lytic *vs*. temperate).

**Conclusions:**

We can thus condense, in relatively simple figures, this phage information dispersed over many publications.

## Background

Bacteriophages are the most abundant biological entities on Earth and their total population is estimated at approximately 10^31 ^particles on earth [1]. In comparison with the estimated 10^30 ^bacterial cells in the biosphere [2], there are thus 10 virus particles for each putative host [3,4]. In aquatic or terrestrial samples, 10^6 ^to 10^7 ^viral particles per milliliter of water or gram of soil are regularly reported. Moreover, these viruses are highly dynamic, leading to approximately 10^23 ^infections per second [5]. The study of phage diversity is crucial for understanding an ecosystem. For instance, the concept of "killing the winner" has been proposed to explain how phage propagation can control host diversity and abundance [6].

Bacteriophages also participate in the evolution of their bacterial hosts. Horizontal transfer of genes from phage to host and vice versa has been well documented [7,8]. Temperate bacteriophages have the capacity to integrate their DNA into that of the host and can also lead to lysogenic conversion in pathogenic bacteria such as *Vibrio cholerae *[9]. Prophages have been shown to contribute to genome diversification [10] and in some environments, the majority of bacteria contains at least one prophage [4,11]. Lawrence et al, (2002) [12] calculated an average of 2.6 prophages per free living bacterial cell, some genomes can contain up to 10% of prophage DNA [13].

Since the sequencing of the first complete genome of bacteriophage Φ × 174 in 1977 [14], several characteristics have been established concerning phage genomes. The size of completely sequenced genomes varies between 2435 bp (Leuconostoc phage L5) and 497 513 bp (*Bacillus *phage G). However the size distribution of phage genomes is not homogenous, possibly because of a bias linked to isolation techniques [15]. Phages genomes ranging in size from 30 to 60 kb, the majority belonging to the Siphoviridae, have been the most sequenced (approximately 55% of the total), and small phage genomes (5 to 20 kb) are the second most abundant size range (approximately 27% of the total). An intriguing gap is observed between 80 and 100 kb, with very few complete genome sequences available followed by large genome sequences. The distribution of morphotypes corresponding to the genomes available in the Genbank phage database reflects what has been observed by electron microscopy [16], with a predominance of tailed phages containing double strand genomic DNA. The extraordinary diversity of phages in nature, the dynamism of phage populations and the lack of homology among most phage genes is a recurrent theme. However it is also common to observe an absence of homology among phage genes belonging to phages infecting the same host and therefore likely closely related. As the number of available sequenced phage genomes increases, their mosaic structure is becoming more evident [17-19]. Phage genome mutation rates, combined with recombination leading to genetic mosaicism as well as the lack of an universal gene, analogous to the 16 S rRNA gene, explain why phage classification is based on the nature of the phage nucleic-acid and virion morphology. Family-specific genes such as viral capsid structural genes have been used as taxonomic tools [20]. However, these methods are limited and do not reveal other phage characteristics such as virus-host relationships. Homology-free methods based on the usage of oligonucleotides (sequence signatures) are potentially interesting to try in phage classification. Numerous studies have shown the utility of genomic signatures for different purposes. Dinucleotide frequencies have been used to compare genomic signatures of prokaryotic genomes [21-25] or phage genomes [26]. Methods based on longer oligonucleotides were further developed for the characterization and classification of bacterial species [27]. Local variations of the genomic signature along the sequence of a genome allow the detection of horizontal transfers and pathogenicity islands [28-33] or prophages remnants [34]. More recently genomic signatures were used in an approach to classify virus genomes, and it was observed that, in general, viral genome signatures are close to that of their hosts [35]. Another use of genome sequence signatures, applied to viruses, was to assign environmental genomic fragments either to a known species or to regroup them in new ones [36].

Recent phages metagenomic studies [11,37,38] have reinforced the view that phage genome diversity is extraordinarily high, that phages with dsDNA are predominant in the environment, and that they constitute an enormous "source" of uncharacterized genes. One of the principal questions that remains to be answered is the nature of phage-host relationships in the context of genomic and metagenomic data, such as the phage life cycle (lytic or temperate), morphotype or host range.

In this report, we have used the genomic signatures of phages and their hosts to aid in the understanding of these relationships. Host signatures from four bacterial species infected by a large number of phages has been compared with phage signature. We calculated a "distance" between each phage and its host. We demonstrate that this distance can be used to group the phages and gives indications of the phages growth cycle.

## Results and discussions

### Choice of the phage genomes used in this study

As of January 2009, there were 521 bacterial and archaeal virus genomes available in the Genbank phage database. Among these genomes, 459 are composed of dsDNA and are mainly distributed among the Orders of the Caudoviridae. The 62 remaining genomes contain ssDNA or RNA and correspond to Microviridae, Leviviridae and Inoviridae members.

We examined the 459 dsDNA phage genomes of the database and, where possible we grouped the different phage genomes by host and collected data concerning their morphotype, whether temperate or lytic, and the genome length.

**Figure 1:**
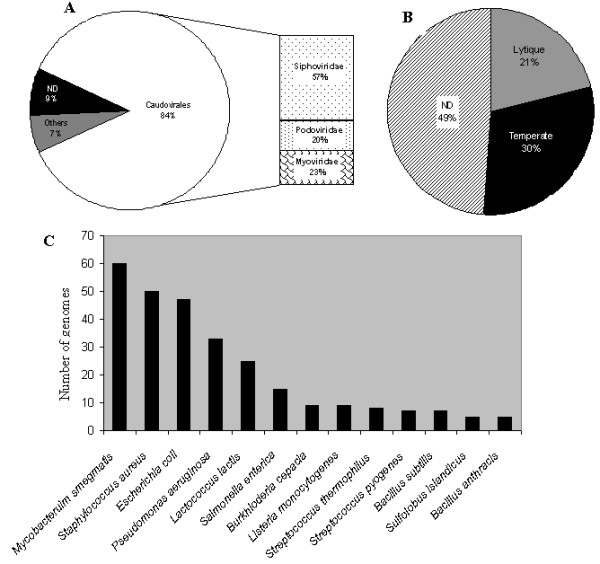
**Distribution of completely sequenced bacteriophage genomes retrieved from Genbank-phage Database**. A: Proportion of genomes belonging to the different phage families. B: Proportion of genomes from temperate or lytic phages. C: Number of completely sequenced genome of phages infecting the same host. Only host with at least 5 different phages are shown. ND: Not indicated in the database.

The Caudoviridae corresponded to 84% of available genomes, composed of 57% Siphoviridae, 23% Myoviridae and 20% Podoviridae families (Figure 1A). This distribution is nearly the same as that published in 2007 concerning the phages examined in the electron microscope [16], although 9% of the available genomes have not been characterized or completely annotated. Approximately one third of the genomes contain an indication of the capacity to lysogenize their hosts. Only 21% have been described as exclusively lytic, whereas for 43% of the phages this information is not mentioned (Figure 1B). The majority (60%) of the genomes available in the database infect only 13 species, with a clear dominance of phages infecting *Mycobacterium smegmatis, Staphylococcus aureus, Pseudomonas aeruginosa and Escherichia coli *(Figure 1C). We thus examined the Caudoviridae members infecting these four bacterial species.

### *Escherichia coli *Caudoviridae

**Figure 2:**
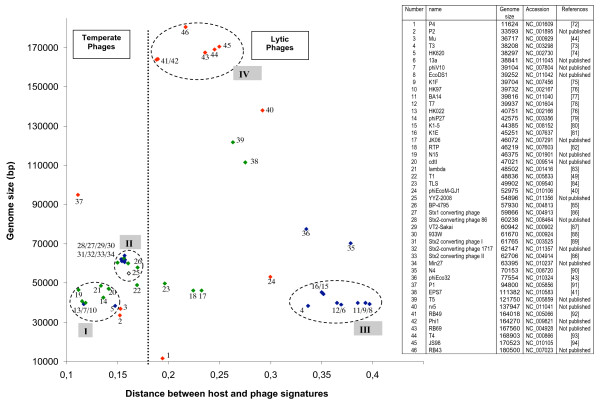
**Distribution of the genomic signature distances of *E. coli *phages as a function of size of phage genomes **[72-94]. Red symbol: Myoviridae, green symbol: Siphoviridae, blue symbol Podoviridae, white symbol: family not indicated. The numbers correspond to the phages listed in the Table.

Forty-six genomes of the order Caudoviridae infecting *E. coli *can be gathered in Genbank phage database. The genomic signature of each phage was generated, as detailed in Methods, compared with the genomic signature of *E. coli *W3110, and the distance between phages and host was calculated. Other *E. coli *strains were tested but the distances were not significantly different (data not shown). The genomic signature distances, morphotypes, genome lengths and life styles are shown in Figure 2.

#### E. coli phage groups

A first feature that is revealed by an analysis of Figure 2 is the coherent grouping of phages. This grouping is in agreement with a 6 groups K-means classification based on phage signatures. The number of groups is greater than those described in Figure 2 to take into account the isolated phages. The groups based on signature distance correspond to the different known and identified groups of coliphages. For example, all the phages belonging to the lytic T7 super-group (group III) have a relatively homogenous distance signature. For temperate phages, two groups can be observed. The first group (group I), containing the lambda-like phages, is characterized by a short distance signature, perhaps reflecting a more ancient prophage life style. The second lambdoid group (II) is very homogenous and contains phages characterized by their ability to carry shiga toxin-like encoding genes. Our representation appears to be compatible with the "classification scheme" suggested by Casjens [39]. The last group (IV) corresponds to the T4 super-group that contains phages with genomes ranging from 164 to 180 kb in length. These genomes have the peculiarity of having a low GC%, necessitating the normalization of genomic signature of the host and phages (see Methods). In spite of the fact that genomes are larger and then likely least host dependent, the overall observed distance is less than that of the T7 super-group. In the *E. coli *phage landscape, several phages remain isolated. Phage ΦEcoM-Gj1 has been recently described and its genome reveals a unique pattern of different origins. It is the first phage with a Myoviridae morphotype but with a T7-like RNA polymerase and a large subunit terminase related to that of phage T1 [40]. Phage EPS7 has been isolated and its genome recently analyzed [41]. This phage belongs to the T5 family and its close genomic signature distance is not surprising. The addition to this group of the phage rv5 is tempting, although rv5 is a Myoviridae. Moreover, the proximity of the T4 super group and the putative T5 group is coherent. Analysis of the T5 sequence by Wang et al (2005) [42] revealed that in the "top 10" homologous phages and genes, RB49, RB69 and T4 are first on the list. Like ΦEcoM-Gj1, phage ΦEco32 has been described as a genome with a large degree of mosaicism [43]. The genomic signature distance of phage N4 seems to allow it to be grouped with ΦEco32, but no genetic relationship can be retrieved from the literature. Finally, phages Mu and P2 show very close distances, whereas Mu is able to integrate as a prophage by a transposition mechanism, while P2 has a site-specific mechanism of genome integration. It is noticeable that significant homology between phage Mu and P2 have been observed for the tail fiber encoding genes [44]. Phage P4, the satellite phage of P2, is a defective phage that exists as a plasmid, shows a more divergent distance signature. Figure 2 confirms that there is no correlation between morphotypes and groups or subtype of phages, although several groups appear to be more homogenous than others. For example, the temperate phage group represented by phage 933W (II) appears more susceptible to exchange modules encoding tail fibers. There is also no significant correlation between genome length and the distance between the host and phage signature. However, our representation, using a combination of the distance signatures, genome length and phage characteristics (life style and morphotype), allows us, independently of sequences comparison, to obtain a coherent picture of the "relationship landscape" of the bacteriophages of *E. coli*.

#### E. coli phage life styles

The second striking observation is the apparent separation between temperate and lytic phages. All the temperate phages are characterized by a host-phage distance ≤0.2. The genomic signature distance seems therefore be sufficiently robust, without any direct sequence comparison, to distinguish these two different life styles. The short genomic distance for temperate bacteriophages is likely due to the long timescale of the "prophage" state. This hypothesis was first suggested by Lawrence and Ochman (1997) [45] to explain that horizontally acquired genes will, over time, adopt the molecular characteristics of the host genome, and has been recently confirmed in a study comparing the sequenced genomes of different strains of the same species [46]. Thus, for temperate phages, the more time a genome remains in a prophage state the smaller should be the genomic signature distance. The difference between temperate and lytic phages of *E. coli *is intriguing because it should not be difficult for a temperate phage to lose its ability to lysogenize its host [39]. The high rate of horizontal transfers in phage genomes is also an argument for the possible acquisition of a functional module involved in lysogeny. The use of genomic signature distances may allow the detection of a temperate phage that has recently lost its lysogenic capacity. In *E. coli*, such examples have not yet been identified, whereas several examples in other species have been reported [47,48]. A lytic phage for which the distance resembles temperate distances is represented by phage T1. In the genome of T1, a homolog of the phage N15 *cor *gene, involved in lysogenic conversion, can be found. When phylogenetic trees are constructed, several lines of descent, including temperate phages such as N15, HK022 and HK97 have been suggested [49]. The largest temperate phage genome P1 shares with N15 the shortest distance. However, the only thing in common between these two phages is a plasmid prophage form, suggesting that the homogenization process between phage and host genomic signatures may be more efficient for plasmids.

### *Staphylococcus aureus *Caudoviridae

Fifty phages of the order Caudoviridae with completely sequenced genomes and infecting *Staphylococcus aureus *were analyzed using the same procedures as described for the *E. coli *bacteriophages.

#### S. aureus phage groups

**Figure 3:**
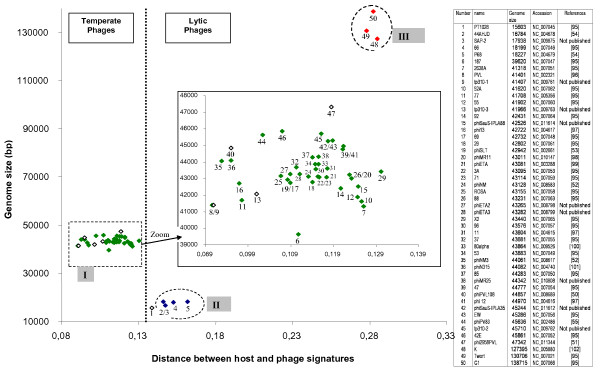
**Distribution of the genomic signature distances of *S. aureus *phages as a function of size of phage genomes**[95-102]. Red symbol: Myoviridae, green symbol: Siphoviridae, blue symbol Podoviridae, white symbol: family not indicated. The numbers correspond to the phages listed in the Table.

Only 8 phages outside of the 39-47 kb genome length range and an average of distances of 0.12 were observed (Figure 3). *S. aureus *strains are often involved in pathogenesis, and represent an important cause of nosocomial infections. Thus, temperate phages with the capacity of lysogenic conversion, such as those containing Panton-Valentine Leukocidin toxins [50-53] are frequently examined. The genomic comparison of 27 phages reported by Kwan et al (2005) [19], based on genome size, nucleotide sequence and proteome comparisons, leads to the description of three separate groups. These 3 groups are retrieved by a K-means classification based on phage signatures and are also clearly evidenced using genomic distances signatures. Group II is composed of lytic Podoviridae with genome sizes inferior to 20 Kb and genomic signature distances around 0.15. Phages 44AHJD and P68 have been classified as Φ29-like by the ICTV, and the presence of a terminal protein at the genome extremities has been confirmed [54]. Phage PT1028 could be assigned to the same group because of its genome size, but no significant homologies can be observed with phages 44AHJD, P68 and 66 [19]. Our results allows us to add phage SAP-2 to group I. Phages K, Twort and G1 (group III) have genomes of approximately 130 Kb, belong to the Myoviridae family, are lytic phages, and have a clearly different signature distance (≅ 0.3) in comparison with the other *S. aureus *phages. The remaining 42 phages were classified in the same group (group I) and contain all the phages of class II, as defined by Kwan et al, 2005 [19]. The highest distance value is observed for phage X2 (0.13) and the smallest value was observed for phage PVL (0.09).

#### S. aureus phage life styles

When information concerning morphotype and life style is available, group I phages belong to the Siphoviridae family and are temperate. It is interesting to note that, as for *E. coli *phages, the temperate phage genomes of *S. aureus *display a tendency to have undergone an amelioration process. The phages that show the smallest distances, PVL, PVL108 and phiPV83 have mutations or insertions that prevent their induction by Mitomycin C [50,51,55]. Phages SLT and 2958PVL possess significant homologies and genome organization with the three "inactive" phages cited above, but their genomic signatures have less resemblance to the host signature.

### *Mycobacterium smegmatis *Caudoviridae

**Figure 4:**
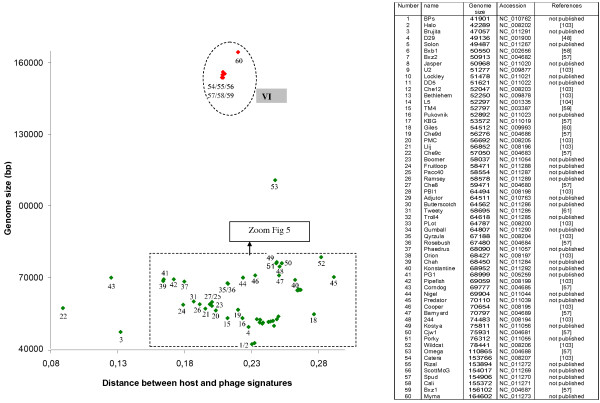
**Distribution of the genomic signature distances of *M. smegmatis *phages as a function of size of phage genomes**[103,104]. Red symbol: Myoviridae, green symbol: Siphoviridae, blue symbol Podoviridae, white symbol: family not indicated. The numbers correspond to the phages listed in the Table.

Sixty completely sequenced genomes of bacteriophages infecting *M. smegmatis *are available in the Genbank phage database. The overall landscape of the Mycobacteriophages obtained with the genomic signature distance (Figure 4) represents the high degree of genetic diversity described using sequence homologies and genome organization methods [18,56]. The distances vary between 0.008 (Che9c) and 0.29 (Predator), with an average (0.22) comparable to that observed in *E. coli *phages.

#### M. smegmatis phage groups

**Figure 5:**
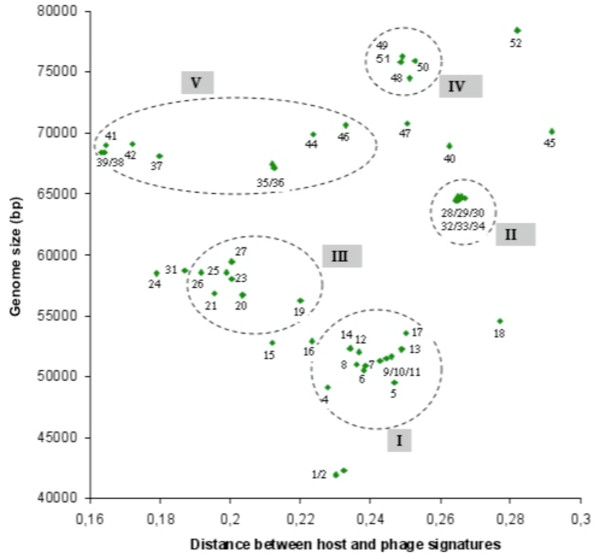
**Zoom of Figure 4 allowing to visualize groups of genomes between 40 and 80 kb**. Red symbol: Myoviridae, green symbol: Siphoviridae, blue symbol Podoviridae, white symbol: family not indicated. The numbers refer to the Table in Figure 4.

Six clusters have been described on the basis of nucleotide similarity [18]. A k-means classification is difficult to perform due to the proximity of small groups of phage (as seen in Figure 4 and 5) that impedes a proper classification. By extrapolation, we have encircled the different clusters, taking as a limit the smallest and the longest genomes. Many phages not yet studied by genome sequence comparison can be added to the different groups. Others, like Omega, Gilles, Predator, Konstantine etc, seem to be more isolated. Group VI, composed only of Myoviridae, is the easiest to discern, whereas to the other group a zoom of the picture is necessary (Figure 5). As observed in *E. coli *and *S. aureus *phages, phage genomes that display significant similarities tend to have similar genomic distance signatures and similar genome size range. For example, cluster V contains phages with significant sequences similarity. However two subgroups are also possible to construct on the basis of genomic signature distances: subgroup A phages number 35, 36, 44 and 46; subgroup B phages number 37, 38, 39, 41, 42 (see table in Figure 4). Indeed, phages of subgroup B show a genomic signature that more closely resembles that of the host. Group II is a very homogenous group for both the genomic signature distance as well as for genome length. Phage Fruitloop appears outside of cluster III but shares a comparable genomic distance signature. The same observation is likely valid for the phages TM4 and Pukovnik that probably belong to group I. In contrast to *E. coli, S. aureus *and *P. aeruginosa *phages, no genomes of Podoviridae infecting *M. smegmatis *have been sequenced, probably because this morphotype (short tail) is not adapted to the complex cell wall of this bacterium [18]. It is clear that, like all other clustering attempts, our representation is unlikely to completely reflect reality, and as more phages genomes infecting the same host become known, better clustering will likely occur.

#### M. smegmatis phage life styles

Contrary to *E. coli, S. aureus *and *P. aeruginosa *phages, the distinction between lytic and temperate life style seems less easy to establish for the different mycobacteriophages. As explained in [57],"most of the phages form plaques with hazy appearance, not obviously either clear or turbid". However stable lysogens can be isolated from these hazy plaques. D29 is a lytic phage very similar to temperate phage L5 [48]. Its status as a lytic phage is due to a 3,6 Kb deletion that removes the repressor. Bxb1 is a temperate phage that forms turbid plaques with a halo, probably due to an enzymatic activity associate with tail particles [58]. TM4 is not considered a temperate phage, thought it was isolated after Mitomicyn C treatment, because no integrase or repressor homolog are present in its genome [59]. Giles and Tweety forms lightly turbid plaques, reflecting a low frequency of lysogeny, but can be considered as temperate because they possess integrases [60,61]. The picture of the genomic distance signatures shows that nearly all the phage genomes are distributed around the average distance. It is interesting to note that Brujita, Che9c and Corndog are more close to their host. Therefore, here we can't propose a "frontier" between lytic and temperate mycobacteriophages. Several hypothesis could explained this fact: (1) all the mycobacteriophages isolated until now are temperate (or are derivatives of temperate like D29); (2) the determination of life style on the basis of plaques morphologies (or the laboratory conditions) is not adapted to the mycobacteriophages; (3) finally we can imagine that these mycobacteriophages have only recently been able to infect *Mycobacterium smegmatis*, or have a different life style as chronic infection, and therefore the amelioration process can't be yet detected by the genomic signature distances.

### *Pseudomonas aeruginosa *caudoviridae

**Figure 6:**
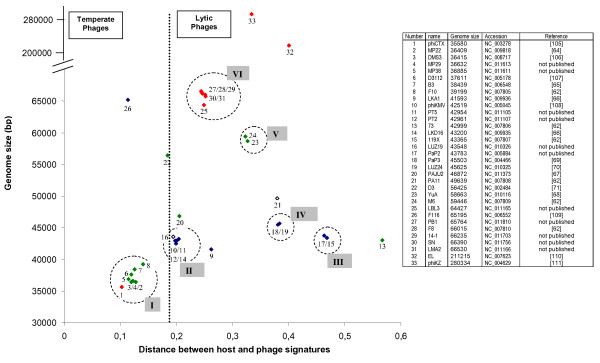
**Distribution of the genomic signature distances of *P. aeruginosa *phages as a function of size of phage genomes **[105-111]. Red symbol: Myoviridae, green symbol: Siphoviridae, blue symbol Podoviridae, white symbol: family not indicated. The numbers correspond to the phages listed in the Table. On the Y axis, a discontinuity was added to accommodate phages 32 and 33.

Thirty-three completely sequenced genomes of phages belonging to the order Caudoviridae and infecting *Pseudomonas aeruginosa *are available in the Genbank phage database. It should be noted that, a significant number of these phages have a %GC significantly lower than that of the host (65%). As seen in Figure 6, although several phages genomes with a GC% that resembles that of the host (e.g. MP22, D3112, B3) show short distances, some others (e.g. YuA, M6) have a similar %GC and a greater distance. In addition, phiKZ, (like T4) has a very low GC% (33%), but the calculated distance is less than that of phage 73 that has a 20% greater GC%. Different hypotheses have been proposed to explain this phenomenon such as the fact that recent horizontal gene transfers in phages infecting hosts with a lower %GC may allow these phages to interact. It is also possible that this large range of %GC is a characteristic of these phages [62]. However, this variation in %GC may also reflect the known high phylogenetic versatility of the *Pseudomonas *genus [63].

#### P. aeruginosa phage groups

As observed for the phages infecting the three other hosts used in this study, it was possible to group the phages as a function of the distance and the genome length (Figure 6). An 8 groups K-means classification is in agreement with this classification. The higher number of groups is due to the isolated phages. Group I is composed of "Mu-like" genomes, but are all Siphoviridae, with the exception of phiCTX. Phage MP22 for example, has been recently sequenced [64] and is highly similar to D3112 except in the gene c and in the late genes of virion morphogenesis. DMS3 has been described has having a high degree of similarity with phage D3112. B3 belongs to the same group of transposable phages and displayed some genetic relationships with D3112 using DNA hybridization [65] and sequence comparisons [62]. MP29, MP38 and F10 are encircled in the same group, whereas F10 presents no significant sequence similarities with B3 and D3112 [62]. The second group is composed of T7 super-group phages, such as phiKMV and LKD16. The unpublished genomes of phages PT5, PT2 and Luz19 are present in the same group. Phages LKD16 and phiKMV present 83% DNA homology with significant differences localized in their early regions [66]. In contrast, LKA1 only show homology at the protein level (48% of the predicted proteins) with phiKMV. Phages 119X, LUZ19 and PaP2 have genomes with very similar length, but only 119x and PaP2 show very similar distances, and the presence of group III is supported by the positive nucleotide comparison between these two phages [62]. Phages LUZ24 and PaP3 are Podoviridae that share 71% nucleotide identities, are grouped, and also share the same genomic signature distance. 24% of the PAJU2 genome is similar to that of phage D3 and 46% of the PAJU2 predicted proteins show similarity with D3 proteins [67], but the nearly 10 kb genome length difference appears to separate them. The last group reported in the literature is the one containing phages M6 and YuA (group V). These two phages share 91% nucleotide identities [68] and have very similar genomic signature distances. Phages LBL3, PB1, F8, 14-1, SN and LMA2 probably constitute another coherent group (group VI). They all show a very homogenous genomic signature distance, the same morphotype and a genome length of 64-66 KB. A Dot-plot genome comparison shows significant nucleotide similarities between these genomes (data not shown).

#### P. aeruginosa phage life styles

The overall landscape of phages infecting *P. aeruginosa *seems less easy to differentiate between lytic and temperate. Indeed the distance observed for the phiKMV group, although higher than the distance observed for the D3112 temperate group, is less different than what is observed for the T7 group of *E. coli*. However, in contrast with the *M. smegmatis *phages, it seems possible to propose a demarcation point separating the lytic and temperate phages, although several atypical cases remain. PaP3, for example, has been described as a temperate phage and LUZ24 as a lytic phage, but their behavior is not totally clear. Indeed, the integration of PaP3 in the host genome has only been demonstrated by restriction enzyme analysis [69], and no indication of immunity or reactivation of an integrated PaP3 prophage is available. On the other hand, LUZ24 forms clear plaques on 36 different strains of *P. aeruginosa*, but small and turbid plaques on strain PAO [70]. The distance (0.38) observed for these two phages is more compatible with a distance characteristic of other lytic phages with genome length of the same order (such as the group of *E. coli *phage containing K1E, K1-5). D3 is a temperate phage with a lambdoid organization, and homologies with HK022/HK97 have been established [71]. A putative integrase has also been detected in the genome of PAJU2, and a lysogenic strain has been isolated [67]. Phage YuA has been described as a temperate phage and it possesses a putative repressor and integrase. But, like phage phiJL001 with which significant similarity is observed, isolation of a stable lysogenic strain was not possible [68]. The YuA distance is more characteristic of the other lytic phages, however it is always possible that the capacity of YuA to infect *P. aeruginosa *is recent and that its genome has not yet evolved through an amelioration process. Finally, like *E. coli *phages P1 and N15, F116 shows a very short distance confirming the hypothesis that the amelioration process is more efficient for phages able to lysogenize their hosts in a plasmid form.

## Conclusions

Bacteriophage genome comparisons, without the need to use tools based on sequence homology is possible using genomic signatures. Our analysis and results, present in one picture per host, allow us to group the phages infecting *E. coli, S. aureus, M. smegmatis *and *P. aeruginosa *and to determine their life cycle (temperate *vs*. lytic).

The hypothesis of the "amelioration" process for the genomes of temperate phages is reinforced by our results. Indeed, the majority of the temperate phages display a shorter genomic signature distance between their genome and that of their host than that of the lytic phages. The genomic distance signature can therefore be a useful tool to predict phage life style.

Finally, putative evolutionary groups, for which available data is often dispatched over a fragmented scientific literature, have been identified on the basis of a conserved genomic signature distance for a coherent genome size range. The genomic signature distance could therefore be a useful tool to assign, without homology sequence comparison, a new phage sequence DNA to a known phage group.

## Methods

### 1/DNA sequences

Caudoviridae (dsDNA) viral genomes infecting four bacterial species and their corresponding host sequences were retrieved from GenBank phage database: *Escherichia coli *(46 phage genomes), *Pseudomonas aeruginosa *(33 phage genomes), *Staphylococcus aureus *(50 phage genomes) and *Mycobacterium smegmatis *(60 phage genomes).

### 2/Sequence signatures

The signature of each sequence is defined as the frequencies of all possible tetranucleotides in the two strands of a sequence represented by a vector. The four hosts under study and their respective phages display large differences in base composition: *E. coli *(strain w3110) GC% = 50.8, *P. aeruginosa *(strain PA7) GC% = 66.6, *S. aureus *(strain RF122) GC% = 32.8, *M. smegmatis *(strain MC2-155) GC% = 67.4. Genomic signatures depend on the relative nucleotide proportion within a genome [27]. As phages infecting the same hosts can present a broad spectrum of nucleotide base composition differences, in order to compare their signatures to their host, we standardized the signatures [27]. Assuming that the succession of nucleotides along a sequence follows a random model (a zero order Markov chain; i.e. that the probability of a particular nucleotide depends only on the nucleotide concentration), the probability to observe a given word is the product of the probabilities of its constituent letters. Therefore, we constructed mock signatures based on the genome base composition under consideration. These signatures were subtracted from the genomic signature of the genome studied in order to obtain the standardized signature.

In order to compare genome signatures, we computed the Euclidian distance $\sqrt{\sum_{i}{\left(V_{i}-H_{i}\right)}^{2}}$ between host and virus signatures: where V corresponds to the virus signature and H to that of the host and i indicates the tetranucleotide under consideration.

## Competing interests

The authors declare that they have no competing interests.

## Authors' contributions

PD designed the study, made the experiments and helped in writing the manuscript. CR analyzed the results, drew the figures and wrote the manuscript. MSD helped in analyzing and presenting the results and helped in writing the manuscript. All authors read and approved the manuscript.

We thank the anonymous referee for his comments and suggestions.
